# Acute Inflammatory Response During Neoadjuvant Chemotherapy for Locally Advanced Breast Cancer: A Case Report

**DOI:** 10.7759/cureus.1332

**Published:** 2017-06-10

**Authors:** Xiaolan Feng, Tanya Berrang, John Paul McGhie, Peter Watson, R Petter Tonseth, Pauline T Truong

**Affiliations:** 1 Medical Oncology, University of British Columbia, BC Cancer Agency; 2 Radiation Oncology, University of British Columbia, BC Cancer Agency; 3 Department of Pathology, University of British Columbia, BC Cancer Agency; 4 Department of Functional Imaging, University of British Columbia, BC Cancer Agency

**Keywords:** locally advanced breast cancer, neoadjuvant chemotherapy, neoadjuvant radiotherapy, complete pathological response

## Abstract

We report on a 56-year-old Caucasian female, diagnosed with locally advanced, hormone-receptor-positive, and human epidermal growth factor receptor 2 (HER2)-positive cancer of the left breast. The patient received neoadjuvant chemotherapy with adriamycin/cyclophosphamide (AC) followed by docetaxel/trastuzumab. A partial clinical and radiographical response was documented after four cycles of AC. Approximately one week after the first cycle of docetaxel and trastuzumab, the patient presented with diffuse edema, erythema, and induration involving the entire left breast. The differential diagnoses included infection, inflammatory response/reaction to docetaxel, or cancer progression. After a multidisciplinary review, the decision was made to stop the docetaxel and deliver neoadjuvant radiation treatment concurrent with trastuzumab. Approximately four weeks after radiation therapy completion, the patient underwent a left total mastectomy and axillary dissection, with pathologic complete response (pCR) in the breast and axillary nodal disease. After surgery, systemic therapy was resumed with paclitaxel and trastuzumab, with a plan to start adjuvant endocrine therapy after completion of chemotherapy. We will discuss clinical considerations in the management of the unexpected findings of acute inflammatory response in the breast and nodal regions during neoadjuvant chemotherapy. Associations between intrinsic breast cancer subtype and pCR in locally advanced breast cancer will also be reviewed.

## Introduction

We report a case of human epidermal growth factor receptor 2 (HER2)-positive locally advanced breast cancer (LABC) with an unusual development of acute breast, skin, and soft tissue inflammation after the first cycle of neoadjuvant docetaxel/trastuzumab. Treatment transitioned to radiotherapy concurrent with trastuzumab, followed by surgery with a complete pathologic response (pCR). Considerations in breast cancer management with the unexpected findings of acute inflammation during neoadjuvant therapy will be discussed. 

## Case presentation

**Figure 1:**
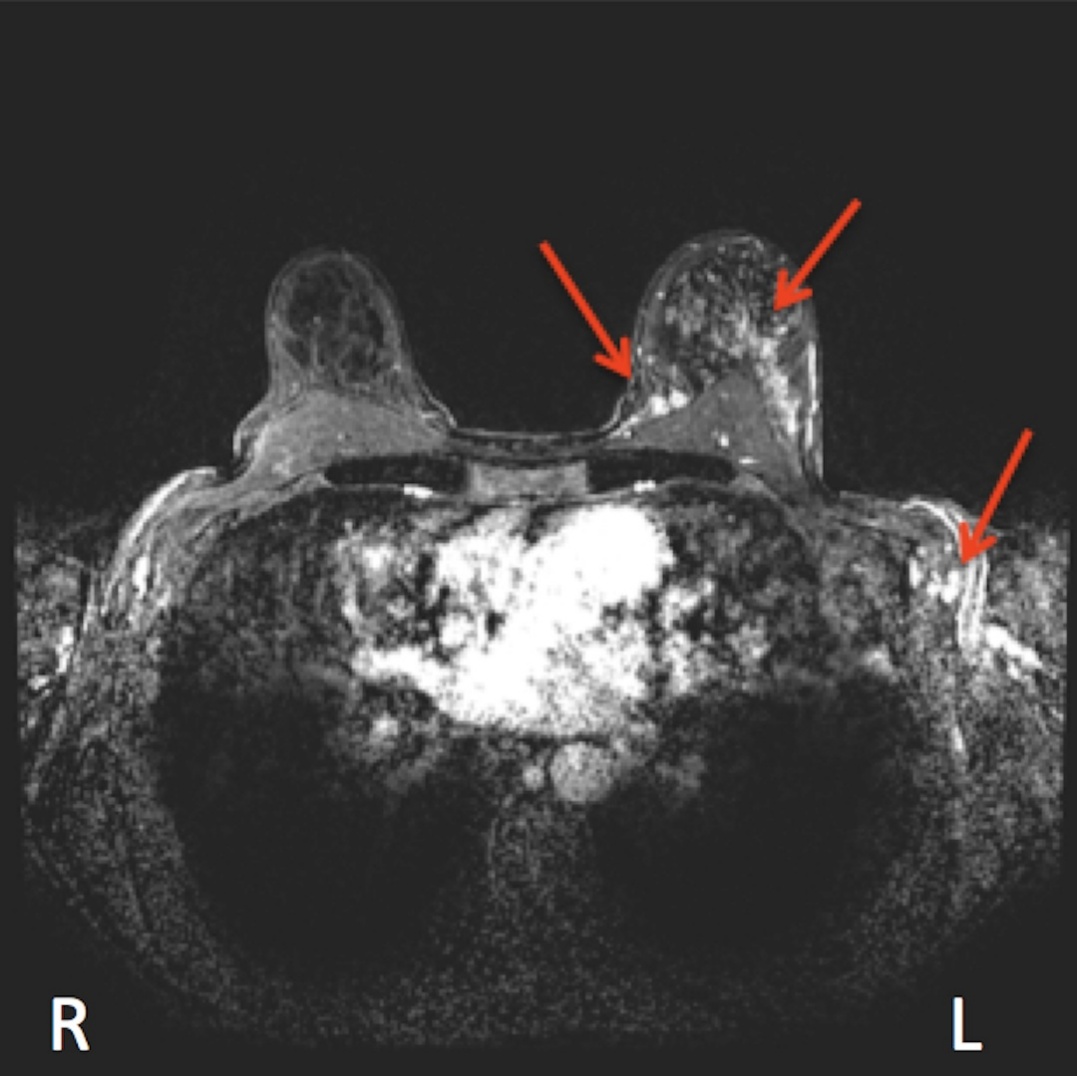
Magnetic resonance imaging before treatment; red arrows indicate multifocal tumors within the breast and multiple left axillary lymphadenopathy

A 56-year-old previously healthy female patient presented with a four-month history of breast heaviness and nodularity. On examination, the entire left breast was firm with diffuse nodularity but no chest wall fixation. There were several non-fixed, non-matted enlarged lymph nodes in the left axilla. The remainder of the examination was unremarkable. Bilateral mammography and breast ultrasound revealed dense fibroglandular tissue with scattered microcalcifications and multiple solid hypoechoic masses involving the left breast. Contrast-enhanced magnetic resonance imaging (MRI) pre-treatment revealed dense fibroglandular tissue with complex signal intensity throughout the left breast, multiple small enhancing nodules in the superomedial quadrant in direct contact with the pectoralis major muscle, and multiple enlarged left axillary lymph nodes (Figure 1).

**Figure 2:**
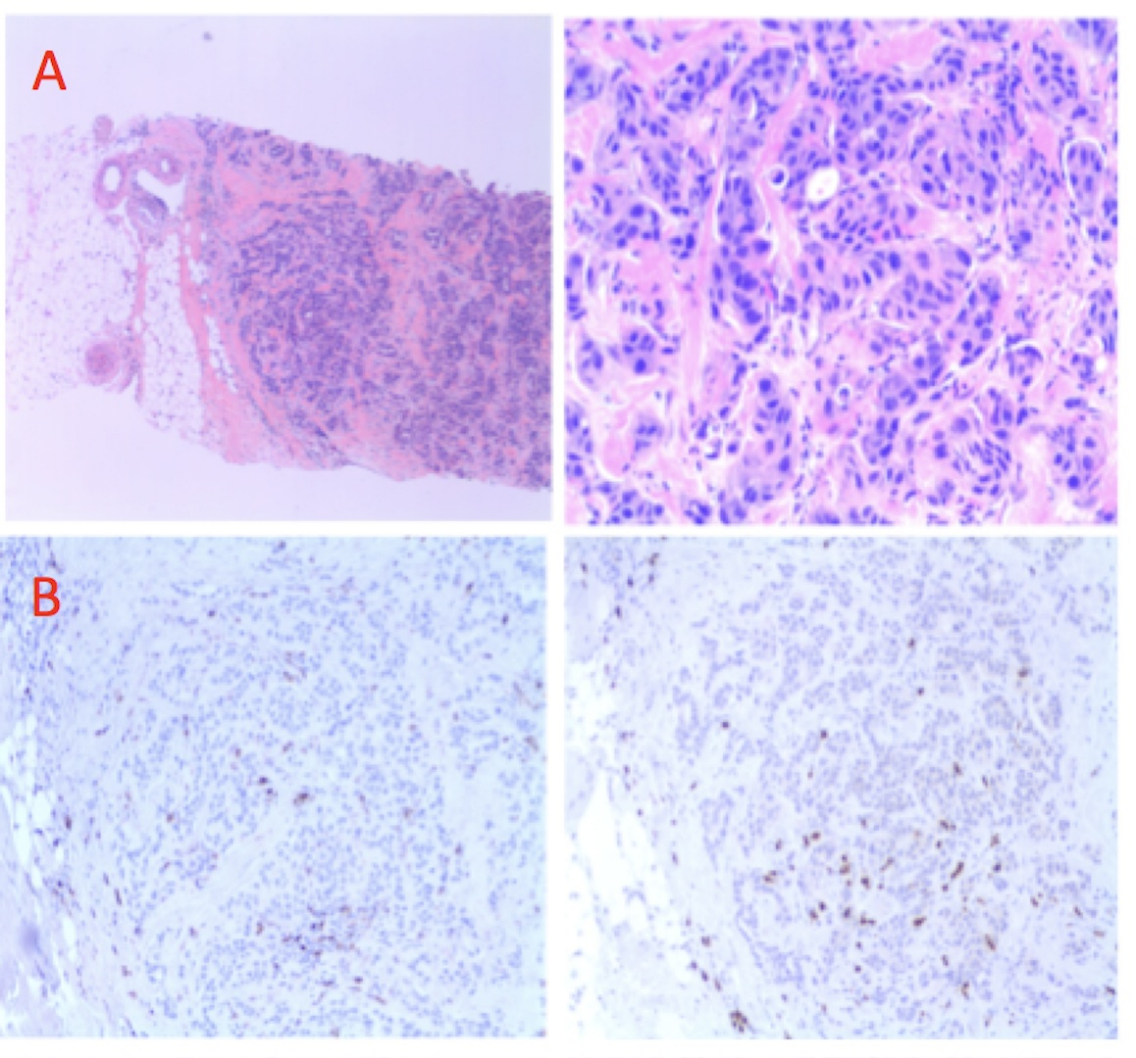
Pathology slides from the ultrasound-guided core biopsies A) Hematoxylin and eosin staining of the breast core biopsy showing invasive ductal carcinoma (Left: low-power 4X and Right: high-power 20X magnificafion); and B) immunohistochemistry staining of tumor in the core biopsy showing relatively sparse tumor infiltrating leucocytes (Left: CD8 and Right: CD163, both images 4X magnification).

Ultrasound-guided core biopsies of the breast (Figure 2A) confirmed invasive ductal carcinoma, Grade III, with no definite in situ disease. Estrogen receptors (ER) were positive (Allred score: 7/8), progesterone receptors (PR) were negative (Allred score: 0/8), HER2 was positive by immunohistochemistry (IHC), and Ki67 was high (25%). Immunohistochemistry was suggestive of low immunoscore on hematoxylin and eosin staining (10%), with sparse stromal and intraepithelial cluster of differentiation (CD) 8 and CD68 infiltrates (Figure 2B), and no expression of programmed death ligand 1.

Staging chest/abdominal computed tomography (CT) and bone scan found no distant metastasis.

**Figure 3:**
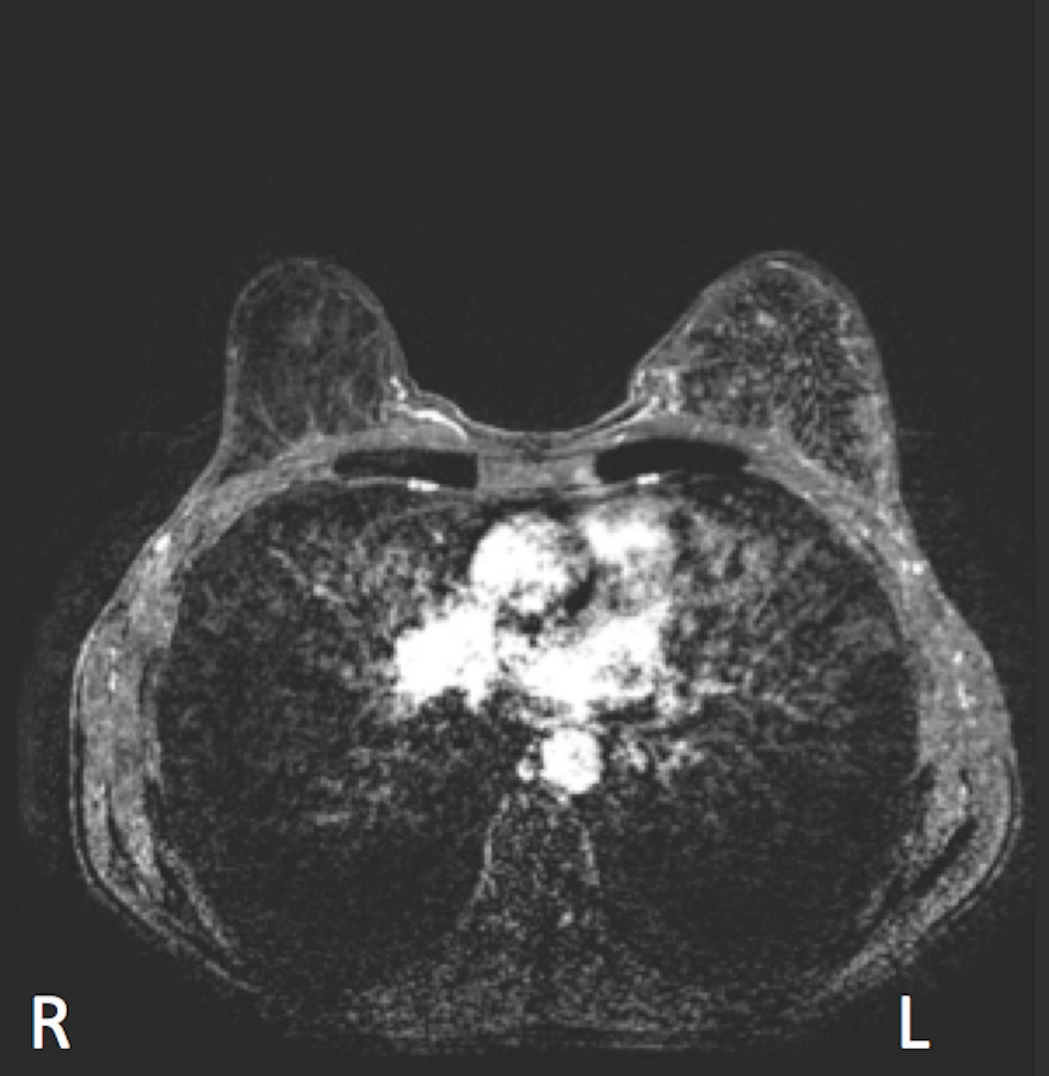
Magnetic resonance imaging showing partial response after three cycles of adriamycin/cyclophosphamide chemotherapy

The patient was evaluated by a breast surgeon, who referred her for oncologic assessment. After multidisciplinary review by a medical oncologist and radiation oncologist, neoadjuvant chemotherapy was recommended with four cycles of adriamycin and cyclophosphamide (AC), followed by four cycles of docetaxel and trastuzumab. There was clinical and radiographical partial response to AC chemotherapy. MRI after three cycles of AC found the nodules in the superomedial left breast and the adenopathy in the left axillary all to be decreased in size (Figure 3).

**Figure 4:**
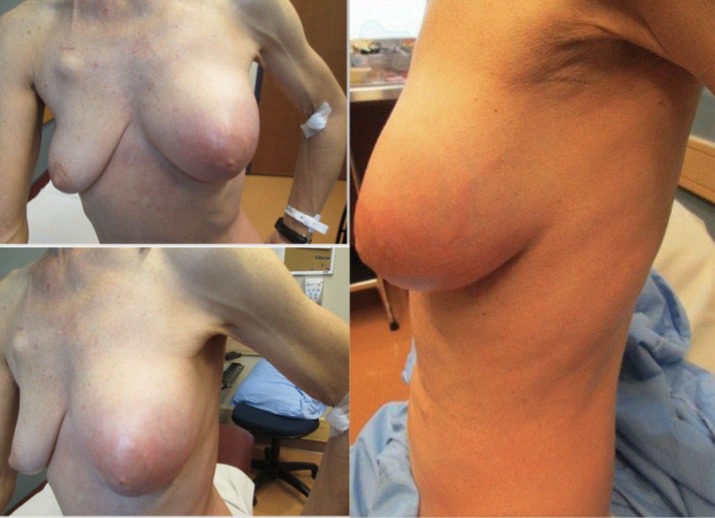
Clinical photographs showing diffuse erythema and swelling involving the breast and surrounding skin and soft tissues after one cycle of docetaxel and trastuzumab

After four cycles of AC chemotherapy, the patient proceeded to the first cycle of docetaxel/trastuzumab on October 25, 2016. Six days later, she developed unexpected swelling of the entire left breast, axilla, and surrounding skin and soft tissue in the left chest. There was breast tenderness and warmth, which increased over the subsequent four days. The patient presented to the emergency room on November 5, 2016. On examination, she was afebrile with normal vital signs and no distress. The entire left breast was diffusely edematous and indurated (Figure 4). There was mild skin erythema, more prominent centrally and at the lower half of the breast, extending from the left chest and flank to the left hip. The overlying skin was tight and stretched by edema, but there were no palpable breast masses. Examination of the left axilla found palpable firm nodes that were not fixed nor matted.

**Figure 5:**
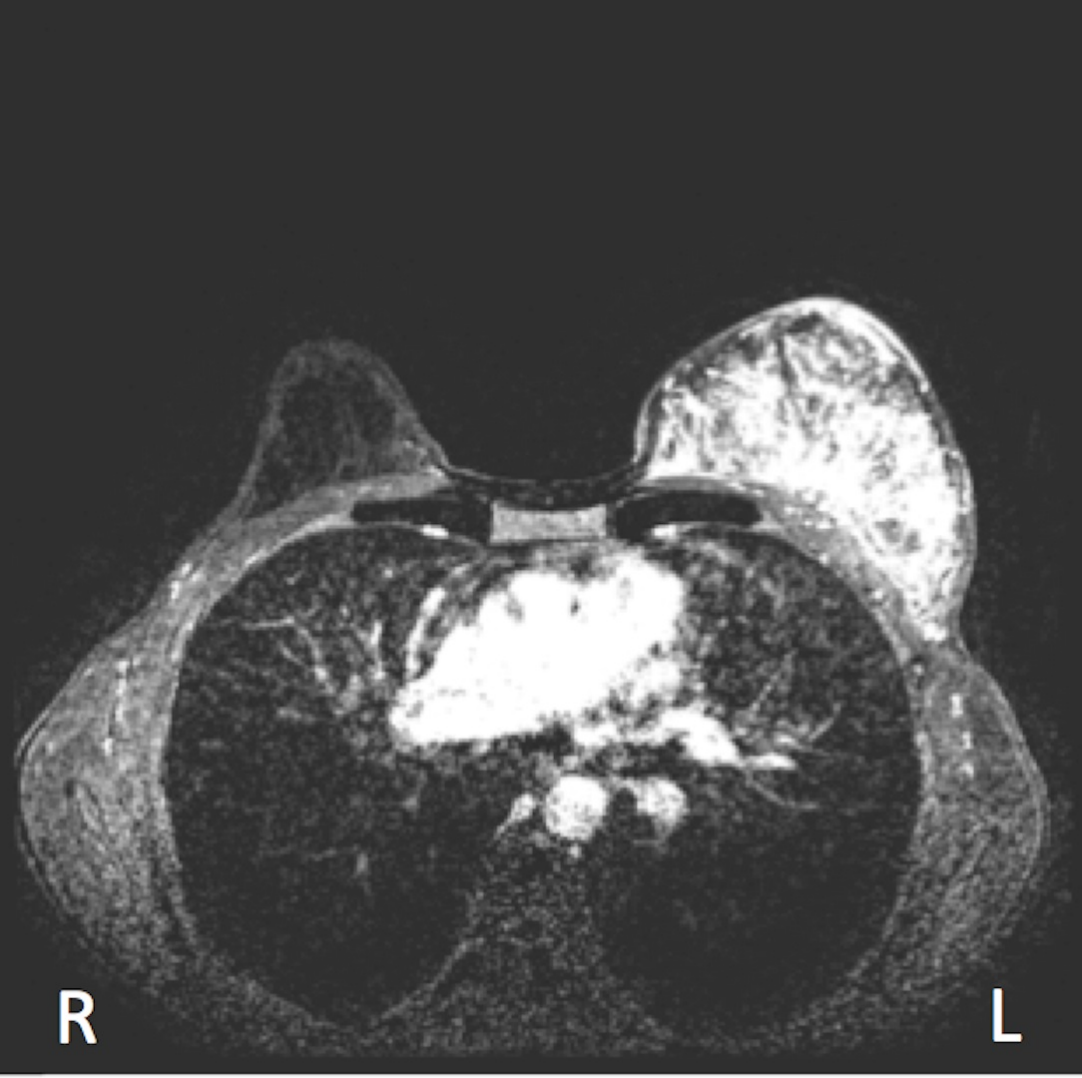
Magnetic resonance imaging showing an inflammatory response in the breast, soft tissues and skin after the first cycle of docetaxel and trastuzumab therapy

The patient was admitted to hospital for further investigation. Although afebrile on admission, she mounted a temperature of 38.4 degrees Celsius that evening. Bloodwork found an elevated white blood count (WBC) at 42 with left-shifted neutrophilia and monocytosis. It was unclear whether the elevated WBC was related to filgrastim or infection/inflammation. Broad spectrum antibiotics were prescribed for presumed mastitis. WBC counts subsequently decreased to 18.7 by November 7 and returned to normal by November 14. Breast ultrasound found no fluid collection, hematoma, or abscess. An MRI done on November 8, 2016 found the left breast to be markedly enlarged with diffuse edema and enhancement but no discrete breast masses or nodules (Figure 5). New lymphadenopathy was identified in the left axilla, possibly representing reactive nodes or metastatic nodal disease.

The case was discussed at a multidisciplinary breast conference. The differential diagnoses included infection, inflammatory response/reaction to docetaxel, or cancer progression. As the breast and overlying skin were highly edematous and distended, surgery was not recommended. The consensus was to discontinue the docetaxel and initiate neoadjuvant radiation therapy concurrent with trastuzumab.

**Figure 6:**
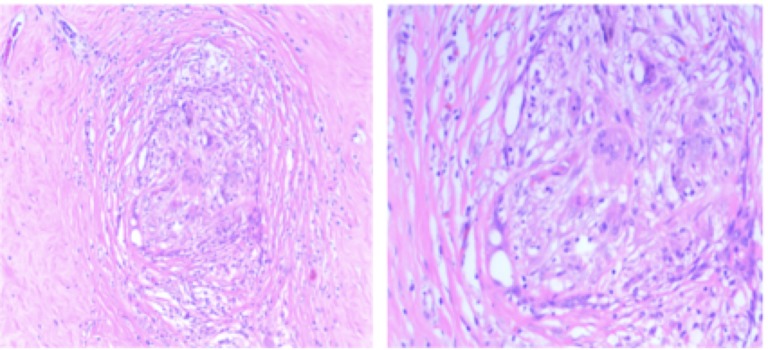
Pathology slides of mastectomy specimen showing stromal fibrosis with no evidence of residual malignancy (Left: low-power 4X and Right: high-power 20X magnification)

The patient received radiation therapy targeting the left breast and the axillary, supraclavicular, and internal mammary nodes to a total dose of 50.4 Gy in 28 fractions. Clinical response was noted with decreased breast size, edema, and erythema within the first two weeks of treatment. Approximately four weeks after radiotherapy completion, the patient underwent a left total mastectomy and axillary dissection removing 11 axillary nodes. Detailed pathology assessment found diffuse stromal fibrosis and reparative changes but no cancer in the breast or axillary nodes (Figure 6).

After surgery, the patient resumed paclitaxel/trastuzumab as adjuvant therapy. The patient has tolerated two cycles well with no adverse effects. The plan is to complete a total of three cycles of adjuvant paclitaxel/trastuzumab. Endocrine therapy will be started after chemotherapy completion and the patient will receive maintenance trastuzumab for a total of 17 cycles.

## Discussion

This case of HER2-positive LABC was interesting due to the unexpected development of acute inflammation of the breast and surrounding tissue after the first cycle of neoadjuvant docetaxel/trastuzumab, raising concerns for tumor necrosis, mastitis, or tumor progression. As the underlying etiology of the acute inflammation was unclear, docetaxel was discontinued and the patient proceeded to radiotherapy concurrent with trastuzumab. This treatment was well tolerated and ultimately enabled prompt surgery, which demonstrated pCR.

It remains unclear whether the inflammatory findings were benign or malignant. The culprit was not trastuzumab as subsequent trastuzumab treatments were well-tolerated with no adverse effects. It is also unlikely that the reaction was related to common docetaxel-induced cutaneous toxicities, such as limb/palmar-plantar erythematous reactions, fixed-plaque erythrodysesthesia, or maculopapular rash [1]. 

While an inflammatory reaction with docetaxel may be encountered in clinical practice, there are few reports describing this effect in the neoadjuvant setting. We speculate that the dramatic inflammatory reaction observed may represent a heightened immune activation as a result of tumor necrosis, contributing to her pCR. The main learning point for breast oncologists is that, in addition to ruling out infectious etiologies, further neoadjuvant systemic and locoregional treatment, such as anti-HER2 and/or radiation therapy, could be considered in such ambiguous situations.

Studies evaluating HER2-positive breast cancer have reported pCR rates ranging between 20% to 40% with neoadjuvant chemotherapy, plus single HER2 blockade, and 40-60% with neoadjuvant chemotherapy, plus combined HER2 blockade [2]. Growing evidence suggests that response to anti-HER2 therapy varies according to hormone receptor status, with lower rates of pCR observed in hormone receptor-positive (luminal HER2), compared to hormone receptor-negative (non-luminal HER2) disease [2].

Factors associated with pCR include lower clinical T stage (T1 vs T3/T4), Grade III histology, and a higher number of cycles of chemotherapy (6-8 vs 3-4) in hormone receptor-positive tumours, negative hormone receptor status, HER2 positivity, and preoperative trastuzumab treatment [2-3]. Neoadjuvant taxane has been reported to double pCR rates and improve disease-free survival in patients with a clinical partial response after AC treatment [4]. Furthermore, progesterone receptor-negative status has been reported to independently predict pCR in HER2-positive tumors [5]. A recent meta-analysis found high Ki67 before neoadjuvant chemotherapy to be associated with higher rates of pCR [6]. Our patient had several of the aforementioned features associated with a higher likelihood of pCR, including clinical T1, albeit multifocal, disease, Grade 3 histology, negative progesterone receptors, high Ki67 index, HER2 positivity, and receipt of preoperative trastuzumab and one cycle of taxane.

The decision to proceed with neoadjuvant radiotherapy and not surgery after the development of significant inflammatory reaction to docetaxel was because the breast and plastic surgeons did not deem that surgery would provide the best oncological and cosmetic outcome at that point. In a population-based analysis of 458 patients with inflammatory breast cancer, Panades, et al. found no difference in clinical outcomes whether the radiotherapy was delivered before or after surgery [7]. A Canadian expert consensus panel advised that patients with clinical findings concerning for disease progression while on neoadjuvant systemic therapy should be reviewed at a multidisciplinary conference to individualize the next management option and that, for patients in whom surgery is not feasible, radiation is a reasonable strategy [8]. Alternatively, one may consider continuing with neoadjuvant chemotherapy but switching to different drugs, another approach supported by some evidence [9].

While pCR may serve as a surrogate marker for an improved long-term outcome, particularly in triple negative and non-luminal HER2 positive tumors [10], controversy remains regarding the prognostic impact of pCR after anti-HER2-based chemotherapy in luminal HER2-positive cases, such as in our case. Some studies suggest that pCR is associated with improved clinical outcome compared to non-pCR regardless of hormone receptor status in HER2-positive breast cancer [4]. Others suggest that pCR is a more suitable surrogate marker in non-luminal HER2-positive compared to luminal HER2-positive disease [10]. As our patient had luminal HER2-positive disease, despite achieving pCR, we plan to complete three cycles of adjuvant paclitaxel/trastuzumab to further eradicate potential distant microscopic disease and to start endocrine treatment after chemotherapy completion.

## Conclusions

An inflammatory reaction involving the breast and surrounding tissues during neoadjuvant chemotherapy may represent tumor necrosis and response to therapy. Ruling out infectious etiologies and continuing neoadjuvant treatment are reasonable approaches. Progesterone receptor negative status, Grade III histology, and a high Ki67 index may be predictive of pCR in luminal HER2-positive breast cancer.
